# First Branchial Cleft Malformation with Duplication of External Auditory Canal

**DOI:** 10.1155/2013/578091

**Published:** 2013-11-07

**Authors:** Pradipta Kumar Parida, Arun Alexander, Kalairasi Raja, Gopalakrishnan Surianarayanan, Sivaraman Ganeshan

**Affiliations:** Department of E.N.T, Jawaharlal Institute of Post Graduate Medical Education and Research, Puducherry 605006, India

## Abstract

First branchial cleft anomalies are uncommon, accounting for less than 10% of all branchial abnormalities. Their rare occurrence and varied presentation have frequently led to misdiagnosis and inadequate and inappropriate treatment of these conditions leading to repeated recurrences and secondary infection. In this paper, a case of 11-year girl with type 2 first branchial cleft defect is described. She first presented with a nonhealing ulcer of upper neck from childhood. Diagnosis had previously been missed and treated as tubercular ulcer. We confirmed the correct diagnosis by history and computerized tomography fistulogram. The lesion was completely excised with no further recurrence.

## 1. Introduction

First branchial cleft anomalies are a special group of congenital malformations of head and neck. The incidence of these lesions is quite low, accounting for fewer than 10% of all branchial cleft anomalies, and often involves the external auditory canal structures [1, 2]. A wide range of clinical manifestations may be observed and they are usually associated with infection. Symptoms in form of cystic swelling, discharging sinuses, and fistulas occur in the periauricular and cervical region located above a line passing through the hyoid bone. Management is often inadequate with recurrences because of misdiagnosis. Several authors proposed classifications to assist appropriate diagnosis and management of these lesions. First branchial arch anomalies have been classified into two types. Type-1 anomaly is of ectodermal origin, manifests as cyst or sinus in the parotid gland, and appears during early or middle adult life [3, 4]. Type 2 defects are ectodermal and mesodermal origin, containing skin with adnexal structures as well as cartilage, and are associated with a sinus/fistula in the anterior triangle of neck, with a communicating tract to external auditory canal (EAC) manifesting during childhood [3, 4]. This paper reports a case of type 2 first branchial cleft defect (collaural fistula) to highlight specific diagnostic clinical features and discusses the significance of this condition in otolaryngology. The roll of computerized tomography (CT) fistulogram in diagnosis type 2 defect of first branchial cleft is described.

## 2. Case Report

An 11-year girl presented to our department with a nonhealing ulcer in right upper neck and intermittent right ear discharge. The neck ulcer first developed when she was 2 years old and initially started as a swelling which ruptured and formed a fistula with a surrounding ulcer. She had had numerous incision and drainage procedures performed for recurrent infections. She also complained of one more recurrent swelling behind right ear for the same duration.

**Figure 1:**
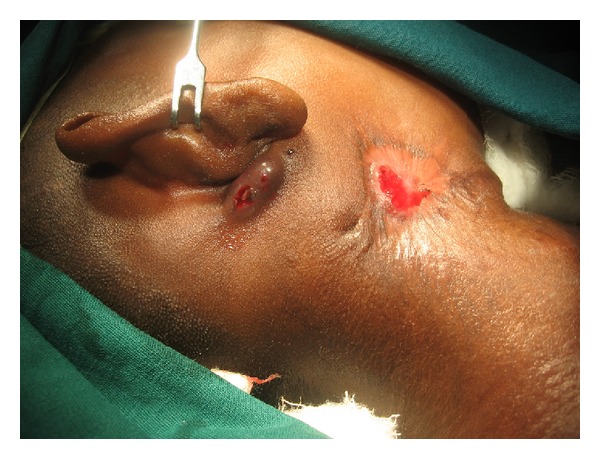
Clinical photograph showing inflammatory opening in right side of neck and a swelling behind the ear.

On examination, there was small cutaneous opening in the right submandibular region surrounded by a 2.5 × 2 cm size ulcer with granulation tissue. The surrounding area was fibrosed and scarred due to repeated surgeries and infection (Figure 1). Ear examination revealed intact tympanic membrane with a small fistulous opening in the floor of right EAC at the bony cartilaginous junction. There was a small swelling of size 2 × 1 cm in the postauricular region (Figure 1).

**Figure 2:**
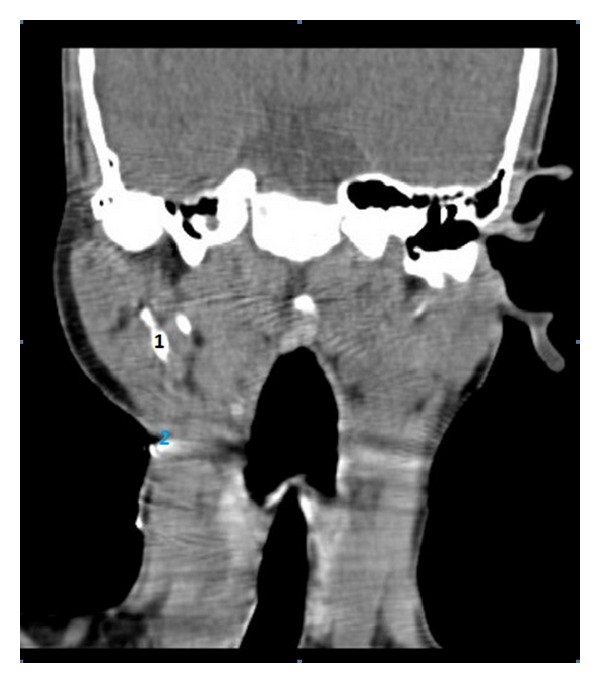
CT fistulogram with coronal reconstruction showing the fistula tract (marked as 1) passing through the deeper part the parotid gland. The cutaneous opening is marked as 2.

**Figure 3:**
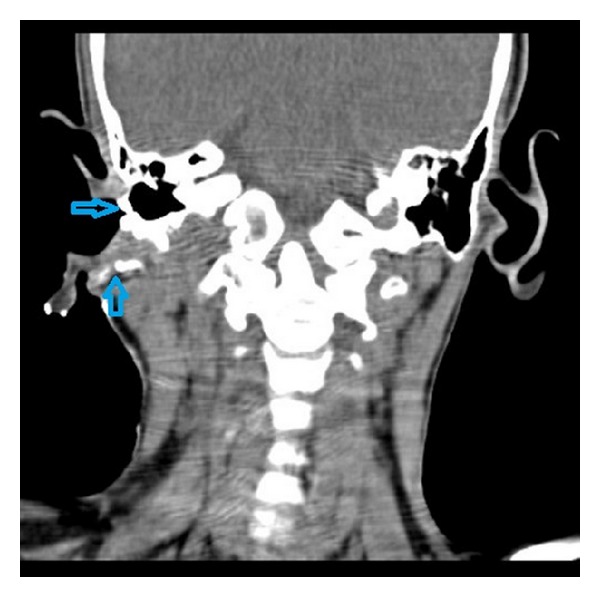
CT fistulogram with coronal reconstruction (posterior to Figure 2) showing the bifurcation of the fistula tract; one opening into the external auditory canal (shown by right facing arrow) and other going to retroauricular region (shown by upward arrow).

**Figure 4:**
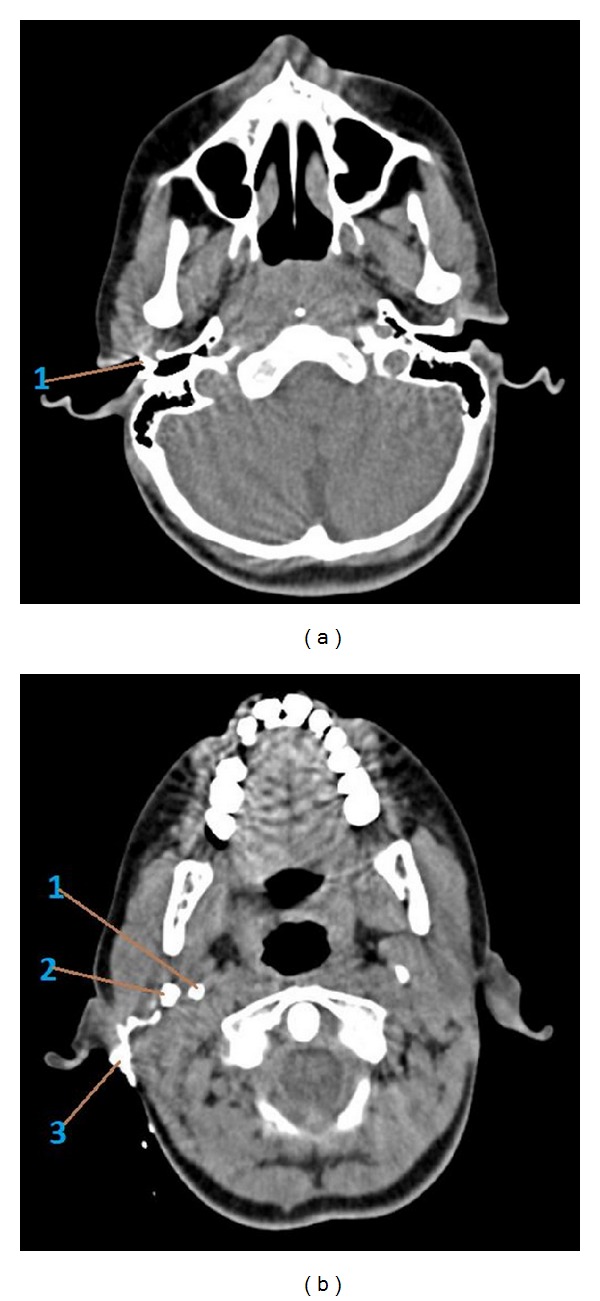
(a) CT fistulogram axial section showing the opening into the external auditory canal at bony cartilaginous junction (marked as 1). (b) CT fistulogram axial section (lower section to Figure 4(a)) showing the bifurcation of the fistula tract. (1) Styloid process, (2) main fistula tract going towards external auditory canal, and (3) bifurcation going to the postaural region with spillage of contrast.

Fistulogram revealed tract starting from skin of right submandibular region ending blindly in the deeper part of parotid gland. Contrast-enhanced computed tomography (CT) showed loss of tissue planes with mild diffuse thickening of submandibular and parotid region. No definite tract could be visualized. As both fistulogram and CT were in conclusive for definitive diagnosis, a CT fistulogram was planned. CT fistulogram was done according to the technique described by Whetstone et al. [5]. The fistula opening in the neck was cannulated with 22-gauge angiocatheter and diatrizoate meglumine (renografin) was infused through the catheter under fluoroscopic visualization. There was prompt filling of the tract. The patient was taken to CT scanner and additional renografin 60 was infused with the patients in scanner. Axial images of neck were obtained and coronal reconstruction was done. Computed tomography fistulogram showed a wide fistulous tract connecting the upper neck to cartilaginous portion of right external ear canal, traversing in a deeper aspect of parotid. The tract showed bifurcation towards the upper end, one part ending in external ear canal and another opening into retroauricular area (Figures 2, 3, and 4). So, a diagnosis of type 2 defect of first branchial cleft was made.

**Figure 5:**
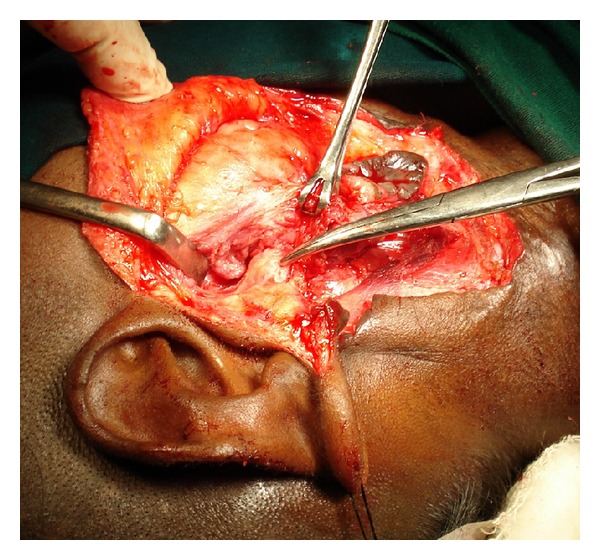
Perioperative photographs showing the thickened aural end of the fistula shown by tip of an artery forceps.

**Figure 6:**
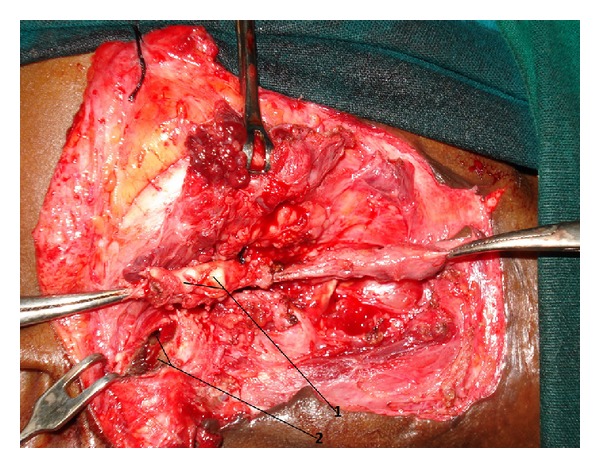
Perioperative photographs after resection of aural end of the fistula. The upper part of the fistula is thick containing cartilage representing the duplication of cartilaginous part of external auditory canal (marked as 1). The defect in external auditory canal is marked as 2.

Complete resection of the fistula tract was performed. Wide exposure of the facial nerve was achieved using modified Blair incision and superficial parotidectomy. During surgical exploration, the fistulous tract was traversing through the parotid gland and noted to run medial to the main trunk of facial nerve. The upper end of the tract was found to be bifurcating, one end ending in floor of EAC at the level of bony cartilaginous portion and the other end going to retroauricular region and ending in a small cyst. The aural end of the tract was thick and containing cartilage while the cervical end was entrapped within the fibrous tissue (Figures 5 and 6).

**Figure 7:**
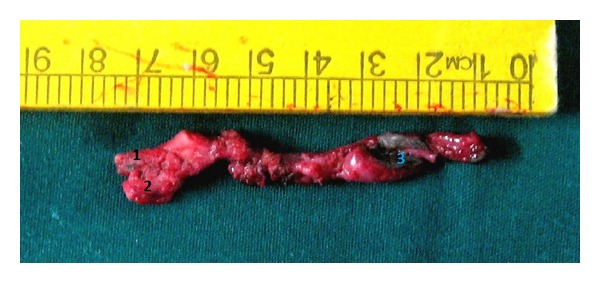
The entire fistula tract after excision. (1) End opening into the external auditory canal, (2) end going to postaural region, and (3) cervical opening with excised ulcer and scarred skin.

The tract was completely excised with a small cuff of EAC cartilage and skin along with the small tract to retroauricular area and surrounding granulation tissue and scar tissue (Figure 7). 

The main branches of facial nerve could be preserved except for marginal mandibular branch, which was embedded in the inflammatory mass of fibrotic tissue that was sacrificed. The defect in EAC was repaired and EAC was packed to prevent stenosis of the ear canal. The skin defect was closed by cervical advancement flap as the primary closure was not possible. The postoperative recovery was uneventful. Histological examination of the excised fistula confirmed that it was lined by squamous epithelium with adnexal structures and cartilage. Thus, the findings of surgical exploration and histopathology confirmed the diagnosis of type 2 defect of first branchial cleft. The patient is asymptomatic with no further recurrence after surgery.

## 3. Discussion

### 3.1. Embryology and Classifications

During the 4th week of human embryological development, 6 pairs of branchial arches appear which are separated by 5 clefts externally and 5 pouches internally. By the seventh week of development, the arches fuse and clefts are obliterated. The structures derived from the first branchial cleft are cavum conchae, the EAC and the external layer of the tympanic membrane [6]. First branchial cleft anomalies are result of incomplete fusion of ventral portion of the first and second arches [6]. During development, closure of the clefts is concurrent with the emergence of developing parotid gland and migration of the facial nerve, which originate from the second branchial arch; thus first branchial cleft anomalies are typically closely related to these structures [6]. The chance of malformations occurring nearer the ear and parotid is greater than that occurring at the hyoid region, as the obliteration of the cleft proceeds from ventral to dorsal. The lesions normally have a close and variable relationship with parotid and facial nerve presumably because of temporal difference during development [7].

Arnot [3], in 1971, proposed type 1 anomalies as defects in the parotid region, appearing during early or middle adult life. Type 2 defects appear in the anterior cervical triangle with a communicating tract to the external auditory canal and usually develop during childhood. In our case, it is type 2 defect by Arnot classification [3]. Work [4], in 1972, proposed a histological classification. Type 1 anomaly is a defect of the ectodermal origin, arising from the duplication of the membranous EAC. They appear as soft cysts lined by squamous epithelium clinically. It can have a tract running medial and parallel to the EAC, superior to the facial nerve and ending in a cul de sac on a bony plate at the level of the mesotympanum. The overlying skin is normal but accidental rupture or secondary infection may result in an intrameatal or a retroinfra-auricular sinus opening. Type 2 defects are ectodermal and mesodermal in origin, containing skin with adnexal structures as well as cartilage. They present as fistula, sinus, cyst, or a combination. They are associated with a sinus or fistulous opening in the region of the submandibular triangle, extending superiorly through the parotid gland towards the floor of the EAC at the level of the bony cartilaginous junction or the cartilaginous portion. In our case it is type 2 defect. Olsen et al. in 1980 classified the defects as cysts, sinuses, or fistulas [1]. First branchial cleft type 2 anomalies are associated with a myringeal web, an epidermal structure which extends from the floor of the EAC to the umbo of the tympanic membrane [8], but it was absent in our case.

### 3.2. Clinical Features

Patients with branchial cleft anomalies most commonly present with swelling in the cervical region (35%), parotid 35%, or periauriclar (24%) region [9]. First branchial cleft anomalies are seen approximately twice as often in woman (69%) as in men (31%) [10]. Fistulas occur more frequently on left side (64%); sinuses show no side preference [10]. First branchial cleft defects are rarely associated with other facial malformations which can contribute to making the correct diagnosis [9]. However, the conditions usually associated with infections and therefore an inflammatory process in region of the Pochet's triangle should immediately raise index of suspicion. Pochet's triangle is the anatomical triangle where first branchial cleft cysts, sinuses, and fistulas were typically located [11]. The limits of the triangle are the external auditory canal above, the mental region anteriorly, and the hyoid bone inferiorly. Otorrhea is the most frequent otological symptoms and the condition should be suspected if recurrent/chronic otorrhea is present in absence of chronic otitis. A sinus or fistula opening in the external auditory canal is present only in 44% of patients, and even if such an opening exists, it may not be necessarily obvious [11]. Analysis of clinical manifestations (cervical, parotid, and auricular) and the findings of careful physical examination focusing on external auditory canal are considered to be more helpful in achieving early diagnosis although fistulous openings into the EAC and myringeal webs are not always present [9].

### 3.3. Investigations

Imaging studies are useful in aiding diagnosis. CT scan can confirm the diagnosis by showing the tract near the external auditory canal [11]. A fistulogram is a useful diagnostic tool in case of a sinus or fistula without signs of cyst formation or inflammation [12]. In present study both CT and fistulogram were inconclusive. Few studies described the utility of CT fistulogram with coronal reconstruction [5, 13] in diagnosis of this condition. The high variability in the course of these lesions and the necessity of complete excision make radiologic visualization of the tract and its bifurcation crucial for proper classification and for surgical treatment. CT fistulogram was found to be more useful and accurate in visualizing and tracing the course of the fistulous tract over CT and fistulogram as in our case. The interesting finding in our case was that the upper end of the tract was divided into two, one was ending in EAC and the other was going to postaural region. This type of CT fistulogram findings is not described earlier.

### 3.4. Management

In first branchial cleft duplication anomalies, misdiagnosis is very common when one is not aware of the possibility of this condition. Triglia et al. noted a delay of 3.5 years between the time of initial presentation and that when adequate treatment was received, with almost 50% of patients having a history of unsuccessful treatment [9]. The resultant recurrent infections and repeated surgery lead to increased scarring and higher risk of iatrogenic facial palsy during its subsequent surgical removal [9]. The course of the fistulous tracts to the facial nerve can be lateral (41%), medial (37%), or between branches (22%) [10]. Out of 10 cases of first branchial cleft anomalies reported by Solares et al. [7], 7 lesions were medial to the facial nerve, 2 were lateral to facial nerve, and 1 was between branches of the facial nerve. Even when the facial nerve is identified and monitored, either temporal (in 18% of cases) or permanent (in 1% of cases) facial nerve injury is a recognized complication [10].

Type 1 cyst can be removed while still keeping the epidermal skin of the meatus intact via a retroauricular incision. Work [4] recommended marsupialisation of this cyst through external auditory canal. For type 2 lesions, early identification of facial nerve at the stylomastoid foramen is recommended [12]. If this part is affected by disease, identifying the facial nerve proximally in temporal bone and tracing it distally may be the safest option. The surgical management for complete removal must always include the possibility of superficial parotidectomy for facial nerve exposure as well as ear operation in combination or alone. Surgical excision of the entire tract and, in some cases, resection of a small amount of skin and cartilage of EAC are the treatment of choice and usually result in permanent cure [14].

## 4. Conclusion

The first branchial cleft malformation may be unrecognized or mistaken for other inflammatory lesions in the periauricular and cervical region. Surgical treatment might then be inadequate leading to recurrence or secondary infection. Complete removal without complications depends on a good understanding of regional embryogenesis, an awareness of the different anatomical presentations, and readiness to identify and protect the facial nerve during resection of the tract. CT fistulogram can be instrumental in minimizing the surgical complications by establishing a definite diagnosis and directing the treatment plan.
